# Use of Japanese Encephalitis Vaccine in US Travel Medicine Practices in Global TravEpiNet

**DOI:** 10.4269/ajtmh.14-0062

**Published:** 2014-10-01

**Authors:** Bhushan R. Deshpande, Sowmya R. Rao, Emily S. Jentes, Susan L. Hills, Marc Fischer, Mark D. Gershman, Gary W. Brunette, Edward T. Ryan, Regina C. LaRocque, Global TravEpiNet Consortium

**Affiliations:** School of Arts and Sciences, Tufts University, Medford, Massachusetts; Department of Quantitative Health Sciences, University of Massachusetts Medical School, Worcester, Massachusetts; Center for Healthcare Organization and Implementation Research, Bedford Veterans Affairs Medical Center, Bedford, Massachusetts; Division of Global Migration and Quarantine, Centers for Disease Control and Prevention, Atlanta, Georgia; Arboviral Diseases Branch, Division of Vector-Borne Diseases, National Center for Emerging and Zoonotic Infectious Diseases, Centers for Disease Control and Prevention, Fort Collins, Colorado; Travelers' Advice and Immunization Center, Massachusetts General Hospital, Boston, Massachusetts; Harvard Medical School, Boston, Massachusetts

## Abstract

Few data regarding the use of Japanese encephalitis (JE) vaccine in clinical practice are available. We identified 711 travelers at higher risk and 7,578 travelers at lower risk for JE who were seen at US Global TravEpiNet sites from September of 2009 to August of 2012. Higher-risk travelers were younger than lower-risk travelers (median age = 29 years versus 40 years, *P* < 0.001). Over 70% of higher-risk travelers neither received JE vaccine during the clinic visit nor had been previously vaccinated. In the majority of these instances, clinicians determined that the JE vaccine was not indicated for the higher-risk traveler, which contradicts current recommendations of the Advisory Committee on Immunization Practices. Better understanding is needed of the clinical decision-making regarding JE vaccine in US travel medicine practices.

## Background

Japanese encephalitis (JE) virus is a mosquito-borne flavivirus that is endemic in much of Asia and parts of the western Pacific. An estimated 70,000 JE cases occur per year; the case-fatality ratio is 20–30%, and 30–50% of survivors have neurologic or psychiatric sequelae.^1^–^3^ For travelers, the risk of disease is generally low but varies with destination, trip duration, season of travel, and planned activities.^2^ Fifty-five travel-associated JE cases were reported in the literature during 1973–2008.^4^

The Advisory Committee on Immunization Practices (ACIP) recommends JE vaccination for all travelers who plan to spend 1 month or more in JE-endemic regions during the transmission season. The ACIP further states that the vaccine should be considered for short-term (< 1 month) travelers going to rural areas whose itineraries or activities place them at increased risk of JE virus exposure, short-term travelers going to areas with known outbreaks, and short-term travelers who are unsure of their itinerary.^2^ Two JE vaccines have been available for use in the United States: an inactivated mouse brain-derived vaccine (JE-VAX, Sanofi Pasteur, Swiftwater, PA) and an inactivated Vero cell culture-derived vaccine (IXIARO, Novartis Vaccines, Cambridge, MA).^2^ JE-VAX was associated with rare but serious hypersensitivity and neurologic adverse events.^2^ Production of JE-VAX was discontinued in 2006, and limited supplies were available until 2011. IXIARO, which is given as a two-dose series administered 28 days apart, was approved by the Food and Drug Administration in March of 2009. Until 2013, IXIARO was licensed for use only in people ages ≥ 17 years.

Few data regarding the use of JE vaccine in clinical practice are available. A 2007 airport survey of US travelers to Asia found that only 11% of higher-risk travelers (defined as those traveling to JE-endemic areas for ≥ 30 days or spending more than one-half their trip in rural areas) had received JE vaccine.^5^ Furthermore, 69% of these higher-risk travelers who had visited a healthcare provider before their trip indicated that JE vaccine had not been recommended during the clinical encounter.

In this study, we describe the demographic characteristics of US residents traveling to JE-endemic countries and evaluate the current use of JE vaccine in a large consortium of US clinical practices that provide pre-travel healthcare. We focused particularly on the time period since the introduction of IXIARO.

## Methods

### Global TravEpiNet clinics.

Global TravEpiNet (GTEN) is sponsored by the Centers for Disease Control and Prevention (CDC), and it is a consortium of US clinical practices that provide pre-travel care to international travelers.^6^ GTEN sites are distributed across the US and include academic practices, healthcare consortia, health maintenance organizations, pharmacy-based clinics, private practices, and public health clinics. An institutional review board at each participating site reviewed and approved the study.

### Study population.

We evaluated international travelers seen at GTEN sites from September of 2009 to August of 2012. Clinicians collected data on travelers using a secure internet tool. For each clinic visit associated with a unique itinerary, travelers provided details about their medical history, destination countries, purpose of travel, geographic type of travel (urban, rural, or both), planned activities, planned accommodations, and duration and dates of travel. Clinicians verified the information provided by travelers and entered additional data on immunization history, health advice provided, vaccines administered, and medications prescribed during the pre-travel encounter. Clinicians were able to review additional geographic details of the itinerary, such as cities or regions visited within a destination country, but these details were not captured further for analysis. If a traveler had an indication for a vaccine according to ACIP guidelines that were current at the time of the clinic visit but the vaccine was not administered, the clinician was required to provide a reason for not administering the vaccine; available options included pre-existing immunity, vaccine not indicated, referred to primary care provider for vaccination, patient declined, medical contraindication, insufficient time, or vaccine not available. Of note, IXIARO was in use throughout the time period of this study; JE-VAX was available only in limited supplies, and all doses had expired by May of 2011.

### Data analysis.

For the purpose of this analysis, we considered the following countries to be endemic for JE in accordance with the CDC definitions^7^: Bangladesh, Brunei, Burma/Myanmar, Cambodia, China, India, Indonesia, Japan, Laos, Malaysia, Nepal, Papua New Guinea, Philippines, South Korea, Sri Lanka, Taiwan, Thailand, and Vietnam. Australia, Bhutan, Mongolia, North Korea, Pakistan, Russia, Singapore, Timor-Leste, and the Western Pacific Islands were excluded from this analysis, because JE cases are very rare or JE risk data are limited. Transmission seasons for each country were defined according to the CDC.^7^ We limited our analysis to travelers ages 17 years or older whose itineraries included travel only to JE-endemic countries. Higher-risk travelers were defined as those traveling for 30 days or longer during transmission season and who were planning to visit a rural setting. Lower-risk travelers were those traveling outside the transmission season, traveling for fewer than 30 days, or only visiting urban settings.

Data analyses were performed using Stata 12.0 (StataCorp, College Station, TX). We used Somers' *D* test and separate random intercept logistic regressions with clinical site as the random effect to evaluate bivariate measures of association. We first performed a bivariate logistic regression analysis of higher-risk travelers to examine the effect of various individual variables on the likelihood that a clinician would deem a JE vaccine as not indicated for a higher-risk traveler who had not previously been vaccinated; the comparison group was higher-risk travelers who received the vaccine. We examined variables included gender, duration of travel (days), time until departure (≥ 30 versus < 30 days from the clinic visit), type of travel (rural versus urban/rural), purposes of travel, and destination countries. In the interest of building a parsimonious model, we included only variables that were significant at a two-sided *P* value of 0.10 in our multivariable random effects logistic regression model. Additionally, we used a two-sided Student's *t* test to examine differences in JE vaccine administration rates between the first and last years of the study.

## Results

During the time period of this study, 29,885 travelers ages ≥ 17 years were seen at GTEN clinics. The median number of travelers seen at each clinic was 488 (interquartile range = 215–1,691). Of 29,885 travelers, 8,289 (27.7%) were planning to travel exclusively to one or more JE-endemic countries, whereas another 2,166 (7.2%) were traveling to JE-endemic countries along with other countries not endemic for JE. For this analysis, we focused only on the former group; we classified 711 (8.6%) as higher-risk travelers and 7,578 (91.4%) as lower-risk travelers (Table 1). Higher-risk travelers were traveling for a median of 50 days (interquartile range = 32–93 days), and lower-risk travelers were traveling for a median of 14 days (interquartile range = 11–21 days). Higher-risk travelers were significantly younger than lower-risk travelers (median age = 29 years versus 40 years, *P* < 0.001). Leisure was the most frequent purpose of travel for both categories of travelers. Travel for research/education, to visit friends and relatives (VFR), and for humanitarian service work represented a greater proportion of higher-risk travel than lower-risk travel, and business travel represented a greater proportion of lower-risk travel. Higher-risk travelers sought pre-travel healthcare earlier than lower-risk travelers (median = 28 days versus 23 days before departure, *P* = 0.004).

Of higher-risk travelers, 11 (1.5%) had received JE vaccine within the previous 2 years, whereas an additional 188 (26.4%) were administered the JE vaccine at their pre-travel visit (Table 1). Of lower-risk travelers, 28 (0.4%) had been vaccinated within the previous 2 years, and 300 (4.0%) were administered the JE vaccine at the pre-travel visit.

Administration of JE vaccine at GTEN clinics increased during the time period of the study. Overall, 7.2% of all travelers who were exclusively visiting JE-endemic countries received the JE vaccine in the first year of the study (September of 2009 to August of 2010) compared with 8.6% of such travelers in the final year (September of 2011 to August of 2012, *P* = 0.04). The proportion of travelers to JE-endemic countries who were deemed higher -risk according to our study criteria did not significantly change over the time period of the study (8.9% in the first year versus 8.3% in the final year, *P* = 0.42).

**Figure 1.**
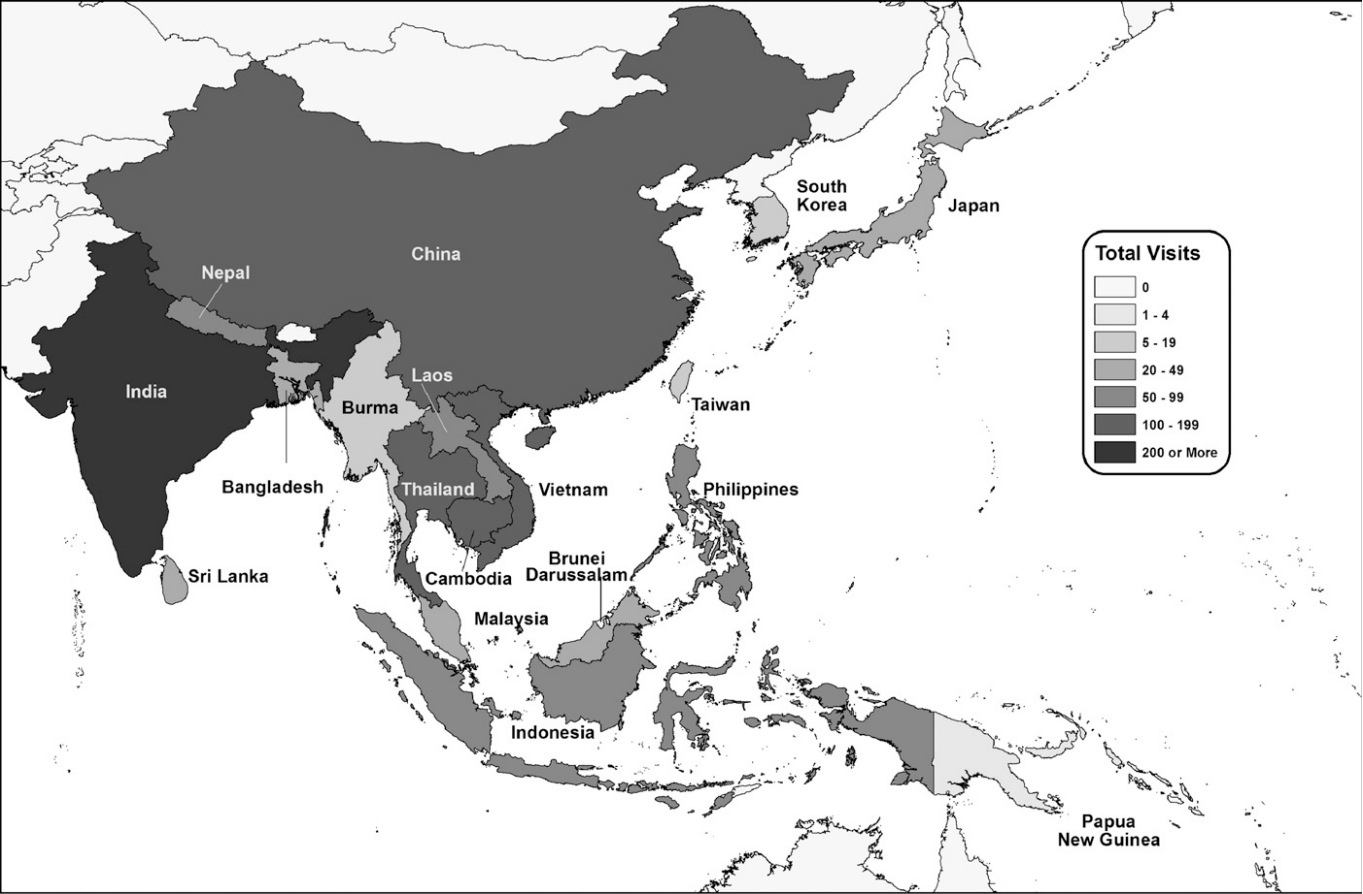
Frequency of visits to JE-endemic countries by higher-risk travelers. Travelers who visited more than one country are included more than one time; 10 travelers to South Korea (8) and Taiwan (2) were excluded because of missing data.

Figure 1 shows the countries visited by higher-risk travelers. Overall, India was the most common destination country for both higher- and lower-risk travelers. The top five destination countries were the same for both higher- and lower-risk travelers and included India (48% of higher-risk travelers and 40% of lower-risk travelers), Thailand (17% of higher-risk travelers and 20% of lower-risk travelers), Cambodia (14% of higher-risk travelers and 18% of lower-risk travelers), Vietnam (13% of both higher-risk and lower-risk travelers), and China (11% of higher-risk travelers and 12% of lower-risk travelers).

In total, 512 (72.0%) higher-risk travelers neither received JE vaccine during the clinic visit nor had been previously vaccinated (Table 1). Table 2 shows the reasons that clinicians provided for not administering JE vaccine to these higher-risk travelers. In the majority of instances (55.1%), clinicians stated that JE vaccine was not indicated for the higher-risk traveler; 116 (22.7%) travelers declined the vaccine, and 85 (16.6%) travelers had insufficient time (< 28 days) to complete the JE vaccine series before departure. Thirty (35.3%) of eighty-five travelers with insufficient time to complete the JE vaccine series were seen by a clinician 14–27 days before their departure date.

We performed a multivariable analysis to identify factors associated with a clinician's determination that the JE vaccine was not indicated for a higher-risk traveler. Clinicians were more likely to consider JE vaccine to not be indicated for VFR travelers and travelers to India (Table 3). Because not all regions of India are endemic for JE, we performed the same multivariable analysis, but we excluded travelers to India; this change did not affect the findings.

## Discussion

Travel to Asia has been increasing in recent decades, placing more travelers at potential risk for JE.^8^ This work is the first description of the use of JE vaccine in a large clinical consortium in the United States since the introduction of IXIARO in 2009. Approximately one-quarter of all travelers in our study were adults who were visiting countries that are endemic or partially endemic for JE. Most of these travelers were considered to be at lower risk for JE according to our classification scheme.

We defined travelers as being at higher risk for JE if they were traveling long-term (30 days or longer) to JE-endemic countries during the transmission season and planning to visit rural areas. ACIP recommends JE vaccination for travelers who plan to spend ≥ 1 month in endemic areas.^2^ However, we found that only slightly more than one-quarter of these higher-risk travelers received JE vaccination in association with their clinic visit. In the majority of cases, JE vaccine was not administered because the clinician reported that the vaccine was not indicated. Clinicians were more likely to consider JE vaccine to not be indicated for VFR travelers and travelers to India.

GTEN sites care for a large number of international travelers, and we speculate that the providers in this study are likely to be familiar with ACIP recommendations for JE vaccine use. This suggests that factors other than lack of awareness of ACIP recommendations influenced their clinical decision-making. Healthcare providers must consider a number of factors when deciding whether to administer a travel-related vaccine, including the details of the traveler's itinerary and risk of exposure to disease, vaccine cost, morbidity and mortality of the disease, and potential adverse events after vaccination.^9^ Although JE can be a fatal disease with high morbidity, the risk of infection for travelers is low.^10^,^11^ A review of published cases of JE in travelers during 1973–2008 estimated the incidence rate for travelers from non-endemic areas to be 0.2 cases per million.^4^ Although current ACIP recommendations are formulated based on these low-incidence rate estimates, clinicians who chose not to administer JE vaccine may have considered this low infection risk in their decision-making. Alternatively, clinicians might have considered the JE vaccine to be too costly, and therefore deemed it not indicated. Country-specific details of the traveler's itinerary that were not included in our analysis may also have influenced the clinicians' risk assessments. Lastly, clinicians may have hesitated to administer JE vaccines given the adverse event profile that was previously associated with JE-VAX. The temporal increase in use of JE vaccine observed in our study suggests that the improved tolerability of IXIARO or increased awareness associated with the licensure of a new vaccine may have influenced clinical decisions.

IXIARO is administered in two doses separated by 28 days.^2^ For approximately one-sixth of higher-risk travelers who did not receive JE vaccine, the clinician indicated that there was insufficient time to complete the vaccine series before departure. A sizable number of these travelers had clinic appointments 14–27 days before departure. Availability of a more accelerated JE vaccine schedule might, therefore, increase the number of travelers who are able to receive the vaccine before departure.

Our study has some limitations. Although GTEN is the largest consortium of clinics providing pre-travel care in the United States, GTEN sites may not be representative of all travel medicine practices. GTEN does not collect data regarding clinician demographics, educational background, or years in practice, and hence, we cannot relate clinician-specific factors to decision-making regarding JE vaccine administration. We also did not collect details on the rationale behind clinical decision-making about JE vaccine, and hence, we cannot determine why clinicians diverged from ACIP recommendations for use of JE vaccine. An in-depth survey of clinician knowledge, attitudes, and practices regarding JE vaccine would be useful for exploring this area.

In summary, we found that many travelers whose itineraries placed them in a category for which JE vaccine is clearly recommended by the ACIP were not offered the vaccine by clinicians, who deemed the vaccine to not be indicated. The failure of clinicians to adhere to practice guidelines is an area in need of additional research.^12^ Clear and accurate information about travel-related disease risks and prevention options needs to be available to healthcare providers and the public. Web-based decision support tools for patients have been useful for improving uptake of other vaccines, such as the measles-mumps-rubella vaccine.^13^ A better understanding of JE vaccine administration practices since the advent of IXIARO is necessary, with a particular focus on understanding why clinical practice differs from current guidelines.

## Figures and Tables

**Table 1:** Demographic and travel-related characteristics of higher and lower JE risk travelers

	Higher-risk travelers (*N* = 711)		Lower-risk travelers (*N* = 7,578)		*P* value*
	*N*	Percent	*N*	Percent	
Age (years)					< 0.001
17–49	569	80.0	5,079	67.0	
50–64	103	14.5	1,728	22.8	
≥ 65	39	5.5	771	10.2	
Gender (female)	400	56.3	3,968	52.4	0.059
Purpose of travel†					
Leisure	351	49.4	4,468	59.0	< 0.001
Research/education	169	23.8	611	8.1	< 0.001
Business	149	21.0	2,174	28.7	0.003
VFR	144	20.3	773	10.2	< 0.001
Humanitarian service work‡	132	18.6	602	7.9	< 0.001
Other§	11	1.6	150	2.0	0.193
Days to departure at clinic visit					0.002
0–13	183	25.7	2,313	30.5	
14–20	89	12.5	1,072	14.1	
21–27	83	11.7	914	12.1	
≥ 28	356	50.1	3,279	43.3	
JE vaccination status					< 0.001
Vaccinated within previous 2 years	11	1.5	28	0.4	
Received vaccine for this itinerary	188	26.8	300	4.0	
Not vaccinated	512	71.6	7,250	95.7	

* *P* value determined using a Somers' *D*-derived test adjusting for clustering among clinical sites.

† Travelers with multiple purposes of travel were included more than one time; therefore, totals sum to > 100%.

‡ Humanitarian service work includes medical service work, non-medical service work, and missionary work.

§ Other reasons include travel to attend a mass gathering, for military purposes, to adopt, or for an explicitly stated other reason.

**Table 2:** Reasons provided by clinicians for not administering the JE vaccine to higher-risk travelers with no previous JE vaccination history (*N* = 512)

Reason	Number	Percent
Vaccine not indicated	282	55.1
Patient declined	116	22.7
Insufficient time	85	16.6
Vaccine not available	11	2.1
Referred to other provider	5	1.0
Medical contraindication	3	0.6
Unknown*	10	2.0

* Data were missing for 10 higher-risk travelers to South Korea (8) and Taiwan (2).

**Table 3:** Adjusted odds ratios and 95% confidence intervals of factors associated with a clinician considering JE vaccination to be not indicated for a higher-risk traveler

Variable*	Odds ratio (95% confidence intervals)	*P* value†
Purpose of travel		
Humanitarian service work	0.59 (0.35–0.99)	0.045
VFR	1.69 (1.03–2.77)	0.039
Business	0.55 (0.33–0.93)	0.026
Research/education	0.50 (0.31–0.82)	0.006
Leisure	1.20 (0.81–1.80)	0.376
Traveling to		
Cambodia	0.73 (0.37–1.45)	0.369
China	1.83 (0.96–3.48)	0.067
India	1.90 (1.22–2.95)	0.005
Japan	0.22 (0.35–1.45)	0.117
Myanmar (Burma)	0.23 (0.22–2.32)	0.211
Thailand	0.99 (0.55–1.79)	0.968

* Travelers with multiple purposes of travel or multiple destinations were included more than one time.

† Obtained from a multivariable random effects logistic regression model.
